# Prediction models and risk assessment for silicosis using a retrospective cohort study among workers exposed to silica in China

**DOI:** 10.1038/srep11059

**Published:** 2015-06-19

**Authors:** Lap Ah Tse, Juncheng Dai, Minghui Chen, Yuewei Liu, Hao Zhang, Tze Wai Wong, Chi Chiu Leung, Hans Kromhout, Evert Meijer, Su Liu, Feng Wang, Ignatius Tak-sun Yu, Hongbing Shen, Weihong Chen

**Affiliations:** 1 Division of Occupational and Environmental Health, JC School of Public Health and Primary Care, the Chinese University of Hong Kong, HKSAR, China; 2 Department of Epidemiology and Biostatistics, Collaborative Innovation Center of Cancer Medicine, School of Public Health, Nanjing Medical University, Nanjing, China; 3 Department of Occupational & Environmental Health and MOE Key lab of Environmental and Health, School of Public Health, Tongji Medical College, Huazhong University of Science and Technology, Wuhan, China; 4 Pneumoconiosis Clinic, Department of Health, HKSAR, China; 5 Institute for Risk Assessment Sciences, Utrecht University, Netherlands; 6 Hong Kong Academy of Occupational and Environmental Health

## Abstract

This study aims to develop a prognostic risk prediction model for the development of silicosis among workers exposed to silica dust in China. The prediction model was performed by using retrospective cohort of 3,492 workers exposed to silica in an iron ore, with 33 years of follow-up. We developed a risk score system using a linear combination of the predictors weighted by the LASSO penalized Cox regression coefficients. The model’s predictive accuracy was evaluated using time-dependent ROC curves. Six predictors were selected into the final prediction model (age at entry of the cohort, mean concentration of respirable silica, net years of dust exposure, smoking, illiteracy, and no. of jobs). We classified workers into three risk groups according to the quartile (Q1, Q3) of risk score; 203 (23.28%) incident silicosis cases were derived from the high risk group (risk score ≥ 5.91), whilst only 4 (0.46%) cases were from the low risk group (risk score < 3.97). The score system was regarded as accurate given the range of AUCs (83–96%). This study developed a unique score system with a good internal validity, which provides scientific guidance to the clinicians to identify high-risk workers, thus has important cost efficient implications.

Silicosis is one of the most important occupational diseases worldwide^1^. It is characterized as a progressive and irreversible lung disease caused by occupational inhalation of dusts. Silicosis is a preventable disease but its prevalence in China has been keeping increasing over the last decade^2^. A total of 23,152 new cases of pneumoconiosis was diagnosed in 2013, accounting for 87.72% of all reported occupational diseases in China, of whom about 35% were confirmed to be silicosis (8,095 cases)^3^. The occurrence of silicosis in China were resulted from prolonged exposures to high levels of silica dust from a variety of industrial activities involving mining, rock drilling, construction activities, steeling rolling, foundry work, and abrasive blasting with silica-containing materials. Despite certain engineering and administrative control of measures (e.g., increasing ventilation, wet process and mask provision) have been adopted in many workplace to reduce workers from the contact of exposure, high occurrences of silicosis and the subsequent premature mortality were frequently observed among tin, tungsten, and gold miners^4^,^5^. Silicosis is an incurable disease with irreversible progressive nature and all treatment strategies are relevant to postponing the progression and control of subsequent medical conditions^6^, mainly from secondary tuberculosis, functional disability, autoimmune diseases, or lung cancer^7^,^8^. The limited value of current treatment toward silicosis leads urgent needs for developing risk prediction models of silicosis for guiding better disease prevention and control among workers exposed to silica dust in China.

Risk prediction of silicosis is an approach of developing statistical models to estimate the probability of developing disease over an adequate time period (latency) that helps clinicians identify workers at higher risk of silicosis, which allows for an earlier counseling of risk factor modifications to decrease risk. Risk prediction model research has been well established in absolute cancer risk prediction, such as breast cancer^9^,^10^,^11^,^12^,^13^; however, it is a relatively new area in the occupational health studies^14^. Although a few of occupational epidemiological studies had explored prediction models to predict the probability of the occurrence of pneumoconiosis or work-related illnesses (e.g., shoulder pain, occupational allergic disease)^15^,^16^,^17^,^18^,^19^, most models were developed based on cross-sectional studies and only the Australia study explored the ‘prognostic model’ to project the occurrence of silicosis and lung cancer in the next 40 years but no discriminatory power was reported^15^.

This study aims to develop a risk model to predict the probability of silicosis occurrence among 3,492 workers exposed to silica dust in China who had been followed up over 33 years. Results from this study provide new insights for risk prediction models among workers exposed to silica dust and thereby offer significant contributions for guiding strategic measures for silicosis prevention in occupational health practice.

## Results

By the end of 2008, we observed 298 incident cases of silicosis with a crude cumulative incident rate of 8.53% after an average of 32.90 ± 8.70 years of follow up with a follow-up rate of 95%, contributing an overall 125,814.69 person-years of observation. As described in Supplementary Table 1, a total of 1,347 workers (38.57%) died and the average age at death was 63.13 ± 12.59 years old. Over 96% of workers were married but more than half of them were illiterate. There were 77.38% workers who had ever smoked (current smoker, 43.44%; former smoker, 33.93%). The average age at first exposure to silica dust was 23.73 ± 6.36 years, while the age at entry into cohort was 27.62 ± 7.81 years, which indicated that some workers had dust exposure prior to their entry into the cohort. The mean concentration of respirable silica dust was 0.08 ± 0.04 mg/m^3^ and the net years of dust exposure was 24.03 ± 9.09 years. Overall, more than half of workers had 2 or more jobs experienced (38.43% had 2 jobs and 23.05% had 3 jobs or more) in the iron ore.

Table 1 presents the results of univariate Cox regression analyses of the potential predictors for the risk of silicosis, including exposure related variables (age at first exposure to silica dust, age of entering the cohort, average exposure to respirable silica dust, cumulative dust exposure time), jobs experienced in the iron ore, socio-demographics (education level, illiteracy; marriage status), smoking habits, and history of lung diseases (pulmonary tuberculosis, chronic bronchitis, and asthma). Six predictors (Table 1) identified to have significant contributions to the risk prediction were age at entry of the cohort (Wald χ^2^ = 155.06, *p* < 0.001), mean concentration of respirable silica dust exposure (Wald χ^2^ = 282.95, *p* < 0.001), net years of dust exposure (Wald χ^2^ = 110.52, *p* < 0.001), no. of jobs experienced in the iron ore (Wald χ^2^ = 43.77, *p* < 0.001), ever smoking (Wald χ^2^ = 5.08, *p* = 0.024), and illiteracy (Wald χ^2^ = 60.75, *p* < 0.001). Results from Schoenfeld residual plots showed that the hazard ratios of all the predictors (Table 1) were likely to be proportional, while the smoothing-spline curves fluctuated around zero; this phenomenon suggested that the assumption of proportionality was satisfied for all the predictors included in the Cox model (data not shown).

We included six significant predictors into the multivariate full Cox model, stepwise selection procedure (0.05 for entry and 0.051 for removing the variables), and the LASSO method for further selection. LASSO model is a shrinkage method which was applied to deal with model’s over-fitting and resulted in a greater reduction in the magnitude of coefficients for the weak predictors than those for the strong predictors. Results of comparing coefficients of models of all predictors (Model1) and two selection methods (stepwise selection procedure, model2; the LASSO method, model3) are summarized in Table 2. Six significant predictors (obtained from the Table 1) were all retained in the Cox model using stepwise selection and LASSO selection approach. Although the coefficient of each selected predictor varied less by using three different selection models, we prefer the LASSO coefficients because the LASSO method reduced coefficients more for the predictors with a weaker association than the stronger ones, and also presented a smallest AIC value (AIC = 4937.97, Akaike information criterion)^20^,^21^.

A bootstrap re-sampling method was applied to generate a large bootstrap dataset^22^, which allows all predictors included in the model becoming statistically significant when 200 bootstraps datasets for LASSO selection was applied. The coefficients of age of entering the cohort, mean concentration of respirable silica dust exposure, net years of dust exposure, no. of jobs experienced in the iron ore, and illiteracy were virtually not affected by the LASSO method, and the results were consistent with their 100% selection in the bootstrap samples. Smoking was selected in 94% of the bootstrap samples. Further analyses revealed that the coefficients for age at first exposure to silica dust, unmarried, pulmonary tuberculosis, chronic bronchitis and asthma were shrunk towards zero, and this provide evidence that the unselected variables (as shown Table 1) had less contributions to the risk prediction of silicosis occurrence. These simulation results showed a clear consistence between the estimated effect of a predictor according to the original LASSO selection and the bootstrapped results.

**Figure 1:**
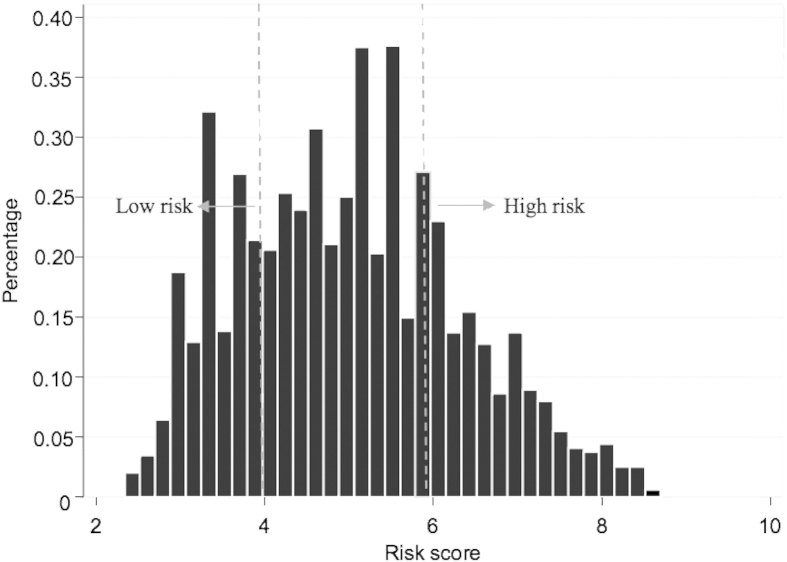
Distributions of risk score of silicosis calculated with a linear combination of the selected predictors weighted by the Cox regression coefficient. Note: low risk group (risk score < 3.97), high risk group (risk score >= 5.91).

**Figure 2:**
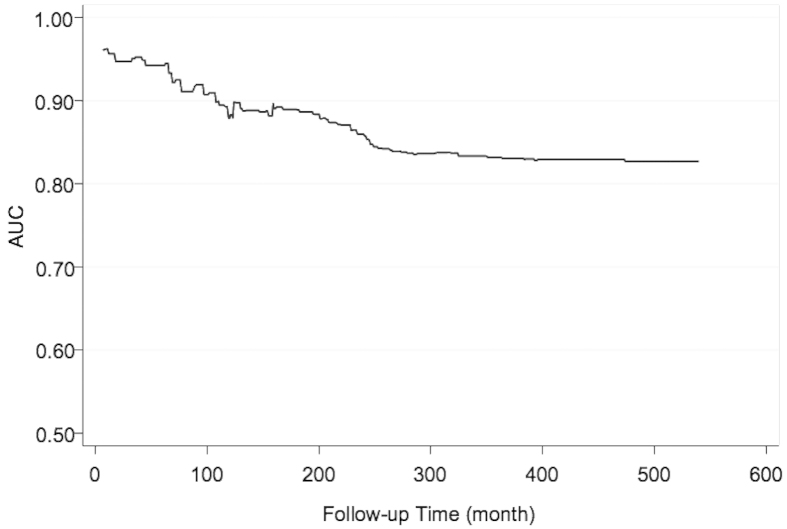
Time-dependent ROC analysis. Figure shows the time-dependent Receiver-Operator Characteristic (ROC) analysis for the risk scores of silicosis along the follow-up time (AUC: Area Under Curves).

Figure 1 demonstrates the results of risk score analysis from the final model by LASSO selection (Model3) incorporating with a linear combination of the six risk predictors weighted by their Cox regression coefficients. The mean of the risk score was 6.53 ± 1.15 and 4.87 ± 1.23 among workers with and without silicosis, with an average score of 5.01 ± 1.31 for the entire all cohort members. The predictive accuracy of this score system was regarded as accurate when it was determined by the time-dependent ROC (Receiver Operating Characteristic) curves (Fig. 2), given an AUC (Area Under the Curve) of more than 0.80 (mean ± s.d.: 0.86 ± 0.40; range: 0.83–0.96) at any time point over the entire follow-up period.

We further split the risk score for silicosis into three subgroups according to its quartiles (Q1, Q3): low risk group (risk score < 3.97), moderate risk group (3.97 ≤ risk score < 5.91), and high risk group (risk score ≥ 5.91). In the iron ore cohort and based on the classification of this score system, 875 workers were classified into the low risk group with 4 (0.46%) silicosis cases, 1,745 workers were classified into the moderate risk group with 91 (5.21%) silicosis cases, and 872 workers were classified into the high risk group with 203 (23.28%) silicosis cases.

## Discussion

This retrospective cohort study with a long follow-up duration (33 years) demonstrated that the risk to silicosis for workers in the iron ore mining could be well predicted based on socio-demographic data and exposure related risk factors according to a newly developed risk score scheme. Our results are thus the novel findings that provide scientific evidence to guide clinicians to differentiate workers at high risk from those at low risk so that targeted prevention can be applied cost efficiently.

The significant predictors ruled in this study are age at the entry of cohort (which stands for age of first exposure to silica dust), smoking, illiteracy, no. of jobs experienced in the iron ore, mean concentration of respirable silica dust exposure, and net years of dust exposure. These findings are consistent with many occupational epidemiological studies that a dose-response relationship was indicated between the occurrence of silicosis and the mean concentration of respirable silica dust exposure or net years of dust exposure^23^,^24^,^25^,^26^,^27^. There is evidence on a synergistic effect between crystalline silica dust and smoking to enhance the risk of silicosis and lung cancer^25^,^27^, and this provides supportive evidence for inclusion of smoking as an independent predictor in our newly developed score scheme to predict the risk of silicosis. On the other hand, age at the first exposure of silica dust might have contributed to the risk of silicosis occurrence, but it was not a significant predictor which was thus not included in our score system. Among 2,396 workers who had past dust exposure prior to the entry of the cohort, the mean dust concentration was quite similar among workers with different age at first dust exposure (Supplementary Table 2A & 2B), which explained the statistical non significance for the relationship between age at first exposure and risk of silicosis, a further possible explanation for this situation should be that those workers were enrolled into this cohort while they were assigned to relatively high-exposure tasks.

Previous studies reported that chronic obstructive pulmonary disease (e.g., chronic bronchitis, emphysema) was associated with a prolonged dust exposure^23^,^24^,^25^,^26^,2^28^,^29^,^30^. A study in Mongolia reported that dust-induced chronic bronchitis and silicosis accounted for 67.8% of occupational diseases^24^. Prolonged exposure to silica dust may lead to airflow obstruction causing chronic bronchitis and emphysema. We included histories of chronic bronchitis diagnosed by doctors as an independent predictor in the Cox model for the risk prediction of silicosis, but it was not statistically significant. It is likely that insufficient cases of chronic bronchitis observed throughout the follow-up period yielded an inadequate power for detection of a significant result based on the context of our current study. On the other hand, biomass burning is an important indoor air pollutant in the remote areas of China, in particular in the early years of follow-up. Exposure to fumes or particular matters emitted from the biomass burning may be associated with the occurrence of respiratory disorders (e.g., chronic bronchitis) or even enhances the risk of silicosis occurrence. We did not collect information on the use of biomass burning, and it thus posed another limitation to the current study.

We developed an unique score system for risk prediction of silicosis using shrinkage estimates with LASSO method in fitting Cox model that enables to adjust for model’s over fitting and avoid extreme predictions^21^; this approach had been applied in a number of case studies^31^,^32^,^33^ but it is less applied in occupational epidemiological studies. We recognized that results from bootstrap re-sampling procedure presented a good internal validity for the LASSO model applied in this study. On the other hand, risk score values developed from our study are evident to have a good predictive accuracy for prediction the occurrence of silicosis in this study (the AUCs ≥ 80%). Moreover, coefficients of the LASSO model transferred to risk score values demonstrated more convenient application for the clinicians in their routine practices and consulting patients at different levels of risk to silicosis, which offers another major advantage of the current study. Nevertheless, there are concerns on the model’s generalizability, as we used cohort data collected from an iron ore to fit the prediction model, which may not represent the whole profile of silica dust exposure workforce in different industrial circumstances. Further validation and calibration of our score system in other industrial settings with large population is warranted.

In conclusion, this study developed a unique risk score system to classify silica dust exposed workers into different risk to silicosis using a LASSO penalized Cox model, with a good internal validity and high model discrimination. This newly developed score system provides clinicians scientific guidance and convenient approaches to identify high-risk workers in routine consultation, thus has important cost efficient implications. Nevertheless, further larger well-designed cohort studies are warranted to validate our results, while cautions should be born in mind when applying the results from our study to other industrial settings.

## Materials and Methods

### The cohort and follow-up

The detailed information for the study design and subjects recruitment has been described in the previous studies^4^. In brief, a total of 3,658 workers who had ever been exposed to silica dust were enrolled in this retrospective cohort from an iron ore mine in China from January 1, 1964 through December 31, 1974 and followed up from 01/01/1964 to 31/12/2008. We included all workers in this cohort with a history of exposure to crystalline silica dust who had worked at least 1 year in the iron ore mining, and excluded workers if she was a female worker (139 individuals), or the male workers who had sufficient evidence of silicosis at the entry of cohort (15 individuals) or he had already died at the entry of the cohort (12 individuals). Eligible subjects finally included in the study were 3,492 workers who had completed information on socio-demographics, lifetime occupational silica dust exposure, and a history of previous diseases diagnosed by the medical doctors. All study subjects provided informed consent and the Research Ethics Committee of Tongji Medical College of the Huazhong University of Science & Technology approved all procedures and all experiments were conducted in accordance with the approved guidelines.

### Diagnosis of silicosis and important predictors

The diagnosis of silicosis was originally made by a diagnostic expert panel comprising of three readers according to the Chinese National Diagnostic Criteria of Pneumoconiosis 1986 (i.e., the routine diagnoses)^34^, hereas all the chest radiographs were re-evaluated by a panel of experts using the Diagnostic Criteria in 2009^35^ and showed a good agreement between the two diagnostic criteria. Following the criteria, silicosis was classified into three stages: stage one, stage two, and stage three, which was very similar with the ILO (International Labour Organization) classification^36^. Silicosis stage one was approximate to the category 1, silicosis stage two was similar to the category 2, and silicosis stage three was equal to the category 3 in concurrence with the appearance of large opacities in ILO classification. Historical industrial hygiene data were collected from the periodical monitoring data for total dust for the period 1964–2008. The total dust concentrations and exposure duration (number of hours) per shift were summarized for each job title in a 3-year interval during 1950–1986 and then measured on an annual basis afterwards. There were 33 job titles confirmed to have exposure to silica dust, number of jobs experienced for workers were included as a measurement for different dust exposure. The concentration of total dust was monitored in the iron ore three times per month by the local industrial hygienists with an overall 55,333 monitored sampling data in total for the period 1964–2008. The average concentration of total dust for each specific job title in a defined calendar year was calculated by taking arithmetic mean of all the samplings of the specific job measured in the corresponding year. It has been noted that workers used half-face respirator as personal protective equipment in the recent decade but only the wet process was applied in the early years of follow-up.

The local industrial hygienist performed the direct measurement for total dust by placing the samplers in a location near workers’ routine practice in a typical working day, and then collected dust samples for a 15–20 minutes interval when the work was in progress according to the Chinese National Sampling Regulations^37^. All samples collected were then weighed to estimate the total dust level in mg/m3; meanwhile the occupational hygienists transformed the total dust level to the concentration of crystalline silica dust for each specific job title according to a validated approach^38^.

### Statistical methods and prediction model specification

We used the Visual FoxPro (VFP) and Microsoft excel software to establish datasets. We then doubly checked the data and cleaned accordingly using SAS software (9.1.3; SAS Institute, Cary, NC). We transformed relevant continuous variables to the categorical scale for clinical use^16^,^17^,^39^. As shown in Supplementary Figure 1, we performed three steps to assess the risk prediction model for the occurrence of silicosis following the principles of Clinical Prediction Model proposed by Ewout W. Steyerberg^21^ **Step I - Predictors selection.** We performed univariate Cox regression analyses to screening out potential predictors by estimating the Hazard Ratios (HR) and their 95% Confidence Intervals (CIs), predictors with *p* < 0.05 will be retained and evaluated further. Three models were performed to check the potential multicollinearity among predictors: full model predictor selection (**Model1**, all predictors entered into the Cox model at the same time), stepwise Cox model selection (**Model2**) and Least Absolute Shrinkage and Selection Operator (LASSO) selection (**Model3**). The LASSO method was used to shrink the coefficients toward zero by setting a constraint on the sum of the absolute standardized coefficients^20^,^40^. Schoenfeld residual plots were used to assess the assumption of proportional hazard ratio according to Harrell’s rho results. If the assumption of proportional hazard ratio was not fulfilled, the extended Cox model was carried out for the development of prediction model^41^,^42^,^43^. **Step II - Risk model construction.** We selected the optimal model from the Step I for risk model construction according to the smallest Akaike Information Criterion (AIC). We then assessed the effect of potential predictors using a risk score analysis on the basis of a linear combination of the selected predictors weighted by the Cox regression coefficient, using a formula as following: 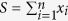, where *S* is risk score, *x* is selected predictors, *n* is the number of selected predictors. **Step III - Risk model evaluation.** Bootstrap re-sampling was used to evaluate the model’s internal validity for the optimal model finally selected^22^,^44^,^45^. The discriminative ability of the model was determined according to the time-dependent Receiver-Operator Characteristic (ROC) curves and the corresponding Area Under the Curve (AUC) were calculated to assess the predictive accuracy of the model; the larger the area is under the AUC, the better the model is for the risk prediction^46^ Sensitivity analyses were conducted to evaluate the performances of different risk scores by plotting (*t*, AUC (*t*)) for different values (cutoff points) of follow up time *t* to test robustness of the results. All statistical analyses were two-sided and performed with R software (Version 3.1.0, 2014-04-10; R Foundation for Statistical Computing).

## Additional Information

**How to cite this article**: Tse, L. A. *et al.* Prediction models and risk assessment for silicosis using a retrospective cohort study among workers exposed to silica in China. *Sci. Rep.* **5**, 11059; doi: 10.1038/srep11059 (2015).

## Supplementary Material

Supplementary Information

## Figures and Tables

**Table 1:** Predictors selection for the prognosis of silicosis using univariate Cox regression model.

**Variables**	**N (%)**	**Coefficient**	**HR (95%CI)**	**Wald*X*^2^**	***df***	***p***	
***Age at first exposure to silica dust (years)***				7.60	3	0.055	
<20	526 (15.06)	0	1.00				
20 ~ 24	654 (18.73)	−0.04	0.96 (0.72—1.28)	0.06	1	0.800	
25–29	1185 (33.93)	0.36	1.43 (1.05–1.96)	5.14	1	0.023	
>=30	1127 (32.27)	0.16	1.18 (0.83–1.68)	0.83	1	0.363	
***Age at entry of the cohort (years)***				155.06	4	<0.001	
<20	606 (17.35)	0	1.00				
20 ~ 24	888 (25.43)	1.26	3.53 (1.36–9.20)	6.69	1	0.010	
25–29	813 (23.28)	2.23	9.38 (3.77–23.32)	23.19	1	<0.001	
30 ~ 34	544 (15.58)	3.06	21.26 (8.64–52.32)	44.25	1	<0.001	
>=35	641 (18.36)	3.30	27.12 (11.09–66.33)	52.34	1	<0.001	
***Mean concentration of respirable silica dust (mg/m^3^)***				282.95	3	<0.001	
<0.05	969 (27.75)	0	1.00				
0.05 ~ 0.10	1715 (49.11)	2.14	8.52 (3.96–18.33)	30.05	1	<0.001	
0.10 ~ 0.15	542 (15.52)	3.41	30.28 (14.08–65.13)	76.17	1	<0.001	
>=0.15	266 (7.62)	4.23	68.64 (31.83–147.98)	116.39	1	<0.001	
***Net years of dust exposure (years)***				110.52	4	<0.001	
<10	332 (9.51)	0	1.00				
10 ~ 15	303 (8.68)	1.21	3.36 (1.91–5.91)	17.58	1	<0.001	
15 ~ 20	367 (10.51)	1.14	3.12 (1.79–5.45)	15.96	1	<0.001	
20 ~ 25	558 (15.98)	1.10	2.99 (1.76–5.11)	16.19	1	<0.001	
>=25	1932 (55.33)	−0.14	0.87 (0.51–1.48)	0.27	1	0.606	
***No. of jobs experienced in the iron ore***				43.77	2	<0.001	
1 job	1345 (38.52)	0	1.00				
2 jobs	1342 (38.43)	0.86	2.37 (1.74–3.23)	30.22	1	<0.001	
3 jobs or more	805 (23.05)	1.06	2.89 (2.09–4.00)	41.28	1	<0.001	
***Smoking habits***							
Non-smoker	790 (22.62)	0	1.00				
Ever smoker	2702 (77.38)	0.35	1.41 (1.05—1.91)	5.08	1		0.024
***Illiteracy***^a^							
No	1734 (49.66)	0	1.00				
Yes	1758 (50.34)	0.13	2.69 (2.10–3.44)	60.75	1		<0.001
***Married status***							
Married	3376 (96.68)	0	1.00				
Unmarried	116 (3.32)	0.14	1.15 (0.64-2.04)	0.21	1		0.645
***History of lung diseases***^b^							
Pulmonary tuberculosis	57 (1.63)	0.16	1.17 (0.52–2.63)	0.15	1		0.097
Chronic bronchitis	207 (5.93)	−0.57	0.56 (0.31–1.03)	3.49	1		0.062
Asthma	34 (0.97)	−0.04	0.97 (0.31–3.01)	0.01	1		0.952

Abbreviation: HR, Hazard ratio; df, Degree of freedom;

^a^Workers whose education attainment were below primary school;

^b^Using workers without a history of lung diseases as the reference group.

**Table 2:** Comparing coefficients of full entry, stepwise and LASSO Cox Regression models for risk prediction of silicosis.

**Regression models**	**Model1**	**Model2**	**Model3**
***Age at entry of the cohort (years)***			
<20 y	ref.	ref.	ref.
20 ~ 24 y	0.97*	0.97*	0.69
25–29 y	1.43**	1.43**	1.17
30 ~ 34 y	1.92***	1.92***	1.66
>=35 y	2.13***	2.13***	1.87
*Test for trend*	0.44***	0.44***	0.43
***Mean concentration of respirable silica dust exposure (mg/m^3^)***			
<0.05	ref.	ref.	ref.
0.05 ~ 0.10	1.43***	1.43***	1.31
0.10 ~ 0.15	2.51***	2.51***	2.38
>=0.15	3.26***	3.26***	3.13
*Test for trend*	1.00***	1.00***	1.00
***Net years of dust exposure (years)***			
<10	ref.	ref.	ref.
10 ~ 15	1.33***	1.33***	1.24
15 ~ 20	1.42***	1.42***	1.33
20 ~ 25	1.65***	1.65***	1.56
>=25	1.04***	1.04***	0.94
*Test for trend*	0.12***	0.12***	0.12
***No. of job experienced in the iron ore***			
1 job	ref.	ref.	ref.
2 jobs	0.61***	0.61***	0.58
3 jobs or more	0.62***	0.62***	0.60
*Test for trend*	0.34***	0.34***	0.33
***Smoking habits***			
Non-smoker	ref.	ref.	ref.
Smoker	0.56***	0.54***	0.53
*Test for trend*	0.58***	0.58***	0.57
***Illiteracy***^a^			
No	ref.	ref.	ref.
Yes	0.38**	0.38**	0.38
*Test for trend*	0.37**	0.37**	0.37

Abbreviation: Model1, Cox model with all predictors entered at the same time; Model2, Cox model with stepwise selection (selection entry *p* = 0.05, removing *p* = 0.051); Model3, Cox model with LASSO selection;

*p value < 0.05; ** *p* value < 0.01; *** *p* value < 0.0001.

^a^Workers whose education attainment were below primary school;

## References

[b1] Greenberg M. I., Waksman J. & Curtis J. Silicosis: a review. Dis Mon 53, 394–416 (2007). 17976433 10.1016/j.disamonth.2007.09.020

[b2] Zhang M., Wang D., Zheng Y. D., Du X. Y. & Chen S. Y. [Analyses on the characteristics and the trends of pneumoconiosis notified between 1997 and 2009, in China]. Zhonghua Lao Dong Wei Sheng Zhi Ye Bing Za Zhi 31, 321–334 (2013). 23803520

[b3] Ministry of Health of China. National occupational diseases report for 2013. (2014). Available at: http://www.nhfpc.gov.cn/jkj/s5899t/201406/ed8ed220d0b74010bcb6dcd8e340f4fb.shtml (Accessed: June 2014)

[b4] Chen W. *et al.* Risk of silicosis in cohorts of Chinese tin and tungsten miners, and pottery workers (I): an epidemiological study. Am J Ind Med 48, 1–9 (2005). 15940718 10.1002/ajim.20174

[b5] Tse L. A., Li Z. M., Wong T. W., Fu Z. M. & Yu I. T. High prevalence of accelerated silicosis among gold miners in Jiangxi, China. Am J Ind Med 50, 876–880 (2007). 17948247 10.1002/ajim.20510

[b6] Leung C. C., Yu I. T. & Chen W. Silicosis. Lancet 379, 2008–2018 (2012). 22534002 10.1016/S0140-6736(12)60235-9

[b7] Oxman A. D. *et al.* Occupational dust exposure and chronic obstructive pulmonary disease. A systematic overview of the evidence. Am Rev Respir Dis 148, 38–48 (1993). 8317812 10.1164/ajrccm/148.1.38

[b8] Yu I. T. *et al.* Further evidence for a link between silica dust and esophageal cancer. Int J Cancer 114, 479–483 (2005). 15578719 10.1002/ijc.20764

[b9] Claus E. B. Risk models used to counsel women for breast and ovarian cancer: a guide for clinicians. Fam Cancer 1, 197–206 (2001). 14574179 10.1023/a:1021135807900

[b10] Santen R. J. *et al.* Critical assessment of new risk factors for breast cancer: considerations for development of an improved risk prediction model. Endocr Relat Cancer 14, 169–187 (2007). 17639036 10.1677/ERC-06-0045

[b11] Xu J. *et al.* Estimation of absolute risk for prostate cancer using genetic markers and family history. Prostate 69, 1565–1572 (2009). 19562736 10.1002/pros.21002 PMC2793526

[b12] Dai J. *et al.* Breast cancer risk assessment with five independent genetic variants and two risk factors in Chinese women. Breast Cancer Res 14, R17 (2012). 22269215 10.1186/bcr3101 PMC3496134

[b13] McCarthy W. J., Meza R., Jeon J. & Moolgavkar S. H. Chapter 6: Lung cancer in never smokers: epidemiology and risk prediction models. Risk Anal 32 Suppl 1, S69–84 (2012). 22882894 10.1111/j.1539-6924.2012.01768.x PMC3485693

[b14] Wilson P. W. *et al.* Prediction of coronary heart disease using risk factor categories. Circulation 97, 1837–1847 (1998). 9603539 10.1161/01.cir.97.18.1837

[b15] Nurminen M., Corvalan C., Leigh J. & Baker G. Prediction of silicosis and lung cancer in the Australian labor force exposed to silica. Scand J Work Environ Health 18, 393–399 (1992). 1336621 10.5271/sjweh.1565

[b16] Kuijpers T. *et al.* A prediction rule for shoulder pain related sick leave: a prospective cohort study. BMC Musculoskelet Disord 7, 97 (2006). 17150087 10.1186/1471-2474-7-97 PMC1762015

[b17] Suarthana E., Moons K. G., Heederik D. & Meijer E. A simple diagnostic model for ruling out pneumoconiosis among construction workers. Occup Environ Med 64, 595–601 (2007). 17409183 10.1136/oem.2006.027904 PMC2092564

[b18] Moshammer H. & Neuberger M. Lung function predicts survival in a cohort of asbestos cement workers. Int Arch Occup Environ Health 82, 199–207 (2009). 18408949 10.1007/s00420-008-0322-4

[b19] Suarthana E. *et al.* A diagnostic model for the detection of sensitization to wheat allergens was developed and validated in bakery workers. J Clin Epidemiol 63, 1011–1019 (2010). 20189762 10.1016/j.jclinepi.2009.10.008

[b20] Tim Hesterberg N. H. C. & Lukas Meier Chris Fraley. Least angle and ℓ1 penalized regression:A review. Statistics Surveys 2, 61–93 (2008).

[b21] EW. S. Clinical prediction mod brels: a practical approach to development, validation, and updating. (eds Gail M. *et al.* ) Ch. 4, 53–81. (Springer, 2009).

[b22] Sauerbrei W. & Schumacher M. A bootstrap resampling procedure for model building: application to the Cox regression model. Stat Med 11, 2093–2109 (1992). 1293671 10.1002/sim.4780111607

[b23] Humerfelt S., Eide G. E. & Gulsvik A. Association of years of occupational quartz exposure with spirometric airflow limitation in Norwegian men aged 30–46 years. Thorax 53, 649–655 (1998). 9828850 10.1136/thx.53.8.649 PMC1745297

[b24] Lkhasuren O., Takahashi K. & Dash-Onolt L. Occupational lung diseases and the mining industry in Mongolia. Int J Occup Environ Health 13, 195–201 (2007). 17718177 10.1179/oeh.2007.13.2.195

[b25] Rego G. *et al.* High prevalence and advanced silicosis in active granite workers: a dose-response analysis including FEV1. J Occup Environ Med 50, 827–833 (2008). 18617839 10.1097/JOM.0b013e31816a9e77

[b26] Cox L. A. Jr. An exposure-response threshold for lung diseases and lung cancer caused by crystalline silica. Risk Anal 31, 1543–1560 (2011). 21477084 10.1111/j.1539-6924.2011.01610.x

[b27] Erren T. C., Morfeld P., Glende C. B., Piekarski C. & Cocco P. Meta-analyses of published epidemiological studies, 1979-2006, point to open causal questions in silica-silicosis-lung cancer research. Med Lav 102, 321–335 (2011). 21834269

[b28] Neukirch F., Cooreman J., Korobaeff M. & Pariente R. Silica exposure and chronic airflow limitation in pottery workers. Arch Environ Health 49, 459–464 (1994). 7818288 10.1080/00039896.1994.9955001

[b29] Wang X., Yano E., Nonaka K., Wang M. & Wang Z. Respiratory impairments due to dust exposure: a comparative study among workers exposed to silica, asbestos, and coalmine dust. Am J Ind Med 31, 495–502 (1997). 9099350 10.1002/(sici)1097-0274(199705)31:5<495::aid-ajim2>3.0.co;2-t

[b30] Meijer E., Kromhout H. & Heederik D. Respiratory effects of exposure to low levels of concrete dust containing crystalline silica. Am J Ind Med 40, 133–140 (2001). 11494340 10.1002/ajim.1080

[b31] Vach K., Sauerbrei W. & Schumacher M. Variable selection and shrinkage: comparison of some approaches. Statistica Neerlandica 55, 53–75 (2001).

[b32] Moons K. G., Donders A. R., Steyerberg E. W. & Harrell F. E. Penalized maximum likelihood estimation to directly adjust diagnostic and prognostic prediction models for overoptimism: a clinical example. J Clin Epidemiol 57, 1262–1270 (2004). 15617952 10.1016/j.jclinepi.2004.01.020

[b33] Srivastava S. & Chen L. Comparison between the stochastic search variable selection and the least absolute shrinkage and selection operator for genome-wide association studies of rheumatoid arthritis. BMC Proc 3 Suppl 7, S21 (2009). 20018011 10.1186/1753-6561-3-s7-s21 PMC2795918

[b34] Ministry of Health of China. Chinese pneumoconiosis diagnosis criteria (GB5906-1986). (1986). Available at: http://www.safehoo.com/Standard/Trade/Particular/200710/5529.shtml (Accessed: 04th Oct. 2007)

[b35] Ministry of Health of China. Chinese pneumoconiosis diagnosis criteria (GBZ70-2009). (2009). Available at: http://www.moh.gov.cn/ewebeditor/uploadfile/2014/11/20141113152629628.pdf (Accessed: 16th March 2009)

[b36] Guidelines for the use of the ILO International Classification of Radiographs of Pneumoconioses, Revised edition 2011. (2011).Available at: http://www.ilo.org/safework/info/WCMS_108548/lang--en/index.htm (Accessed: 17th Nov. 2011)

[b37] Ministry of Health of China. Specifications for air sampling for hazardous substances monitoring in the workplace (GB159-2004). (2004). Available at: http://niohp.chinacdc.cn/zyysjk/zywsbzml/201210/t20121015_70657.htm (Accessed: 15th Oct. 2012)

[b38] Zhuang Z. *et al.* Estimating historical respirable crystalline silica exposures for Chinese pottery workers and iron/copper, tin, and tungsten miners. Ann Occup Hyg 45, 631–642 (2001). 11718659

[b39] Halbesma N. *et al.* Development and validation of a general population renal risk score. Clin J Am Soc Nephrol 6, 1731–1738 (2011). 21734089 10.2215/CJN.08590910

[b40] Tibshirani R. The lasso method for variable selection in the Cox model. Stat Med 16, 385–395 (1997). 9044528 10.1002/(sici)1097-0258(19970228)16:4<385::aid-sim380>3.0.co;2-3

[b41] Hosmer D. W., Lemeshow S. & May S. Descriptive methods for survival data, Second edition. (eds David J. Balding *et al.* ) Ch 2, 16–66 (Wiley-Interscience, 2008).

[b42] Machin D., Cheung Y. B. & Parmar M. Survival analysis: a practical approach. (eds Gill R. *et al.* ) Ch. 5, 121–155 (John Wiley & Sons, 2006).

[b43] Kleinbaum D. G. & Klein M. Survival Analysis. Ch. 3, 97–157 (Springer, 2012).

[b44] Davison A. C. Bootstrap methods and their application. (eds Gill R. *et al.* ) Ch. 2, 11–66 (Cambridge university press, 1997).

[b45] Denne C., Maag S., Heussen N. & Hausler M. A new method to analyse the pace of child development: Cox regression validated by a bootstrap resampling procedure. BMC Pediatr 10, 12 (2010). 20205739 10.1186/1471-2431-10-12 PMC2837865

[b46] Heagerty P. J., Lumley T., Pepe M. S. & Time-dependent R. O. C. curves for censored survival data and a diagnostic marker. Biometrics 56, 337–344 (2000). 10877287 10.1111/j.0006-341x.2000.00337.x

