# Supplementary material for: Prediction models and risk assessment for silicosis using a retrospective cohort study among workers exposed to silica in China
Source: Sci Rep. 2015 Jun 19;5:11059. doi: 10.1038/srep11059 (PMC4473532; doi:10.1038/srep11059)
Supplement: Supplementary Information [file srep11059-s1.pdf]

Supplementary Materials

Prediction models and risk assessment for silicosis using a retrospective cohort study among workers exposed to silica in China

Lap Ah Tse, Juncheng Dai, Minghui Chen, Yuewei Liu, Hao Zhang, Tze Wai Wong, Chi Chiu Leung, Hans Kromhout, Evert Meijer, Su Liu, Feng Wang, Ignatius Tak-sun Yu, Hongbing Shen, Weihong Chen

Supplementary Figure 1. Workflow of the process of risk prediction model for silicosis

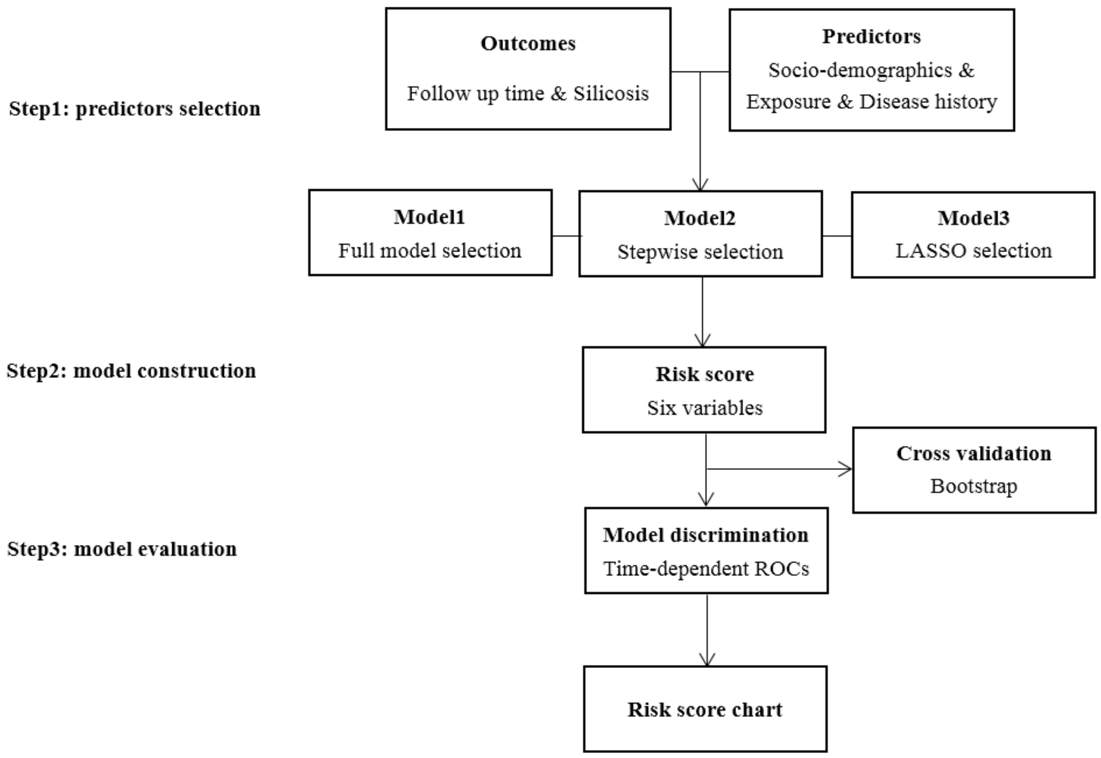

Supplementary Table 1. Basic characteristics of cohort subjects in the iron ore during the period of 1964-2008

Characteristics	N (%) *
Eligible subjects	3492
Alive / Dead	2145 (61.43) / 1347 (38.57)
Age of death (yr.)	63.13 ± 12.59
Years of follow-up	36.03 ± 10.37
Person-years of observation	125814.69
Illiteracy [a]	1758 (50.34)
Married status [b]	3376 (96.68)
Smoking status [c]	2702 (77.38)
Disease history	
Pulmonary tuberculosis	57 (1.63)
Chronic bronchitis	207 (5.93)
Asthma	34 (0.97)
Silicosis patients	298 (8.53)
Age at first exposure to silica dust (yr.)	23.73 ± 6.36
<20	526 (15.06)
20~24	654 (18.73)
25-29	1185 (33.93)
>=30	1127 (32.27)
Age of entering the cohort (yr.)	27.60 ± 7.74
<20	606 (17.35)
20~24	888 (25.43)
25-29	813 (23.28)
30~34	544 (15.58)
>=35	641 (18.36)
Average exposure to respirable silica dust (mg/m^3-yr.)	0.08 ± 0.04
<0.05	969 (27.75)
0.05~0.10	1715 (49.11)
0.10~0.15	542 (15.52)
>=0.15	266 (7.62)
Cumulative dust exposure time (yr.)	24.03 ± 9.09
<10	332 (9.51)
10~15	303 (8.68)
15~20	367 (10.51)
20~25	558 (15.98)
>=25	1932 (55.33)
Job titles in the iron ore	
1 job	1345 (38.52)
2 jobs	1342 (38.43)
3 jobs or more	805 (23.05)

Note: yr.: years; * The continuous variables are expressed as mean ± standard deviation; [a], Workers who had never been to primary school; [b] Married workers; [c] Smokers including current and ever smokers.

Supplementary Table 2A. Summary for mean concentration of respirable silica dust (mg/m³) among different groups for age at entry of the cohort (years) among workers who had a dust exposure before entering the cohort

Groups	Mean	Std. Dev.	Freq.
**<20**	0.09	0.05	722
**20~24**	0.09	0.04	733
**25-29**	0.09	0.04	472
**>=30**	0.09	0.04	469
**Total**	0.09	0.04	2396

Supplementary Table 2B. Summary for mean concentration of respirable silica dust (mg/m³) among different groups for age at first exposure to silica dust (years) among workers who had a dust exposure before entering the cohort

Groups	Mean	Std. Dev.	Freq.
**<20**	0.06	0.03	201
**20~24**	0.07	0.04	436
**25-29**	0.09	0.04	631
**30~34**	0.09	0.05	499
**>=35**	0.10	0.05	629
**Total**	0.09	0.04	2396
